# GLP-1 receptor agonist improves metabolic disease in a pre-clinical model of lipodystrophy

**DOI:** 10.3389/fendo.2024.1379228

**Published:** 2024-04-30

**Authors:** Ahlima Roumane, George D. Mcilroy, Nadine Sommer, Weiping Han, Lora K. Heisler, Justin J. Rochford

**Affiliations:** ^1^ The Rowett Institute and Aberdeen Cardiovascular and Diabetes Centre, University of Aberdeen, Aberdeen, United Kingdom; ^2^ Institute of Molecular and Cell Biology, Agency for Science, Technology and Research (ASTAR), Singapore, Singapore

**Keywords:** lipodystrophy, glucagon-like peptide-1, diabetes, seipin, liraglutide

## Abstract

**Aims:**

Individuals with lipodystrophies typically suffer from metabolic disease linked to adipose tissue dysfunction including lipoatrophic diabetes. In the most severe forms of lipodystrophy, congenital generalised lipodystrophy, adipose tissue may be almost entirely absent. Better therapies for affected individuals are urgently needed. Here we performed the first detailed investigation of the effects of a glucagon like peptide-1 receptor (GLP-1R) agonist in lipoatrophic diabetes, using mice with generalised lipodystrophy.

**Methods:**

Lipodystrophic insulin resistant and glucose intolerant seipin knockout mice were treated with the GLP-1R agonist liraglutide either acutely preceding analyses of insulin and glucose tolerance or chronically prior to metabolic phenotyping and *ex vivo* studies.

**Results:**

Acute liraglutide treatment significantly improved insulin, glucose and pyruvate tolerance. Once daily injection of seipin knockout mice with liraglutide for 14 days led to significant improvements in hepatomegaly associated with steatosis and reduced markers of liver fibrosis. Moreover, liraglutide enhanced insulin secretion in response to glucose challenge with concomitantly improved glucose control.

**Conclusions:**

GLP-1R agonist liraglutide significantly improved lipoatrophic diabetes and hepatic steatosis in mice with generalised lipodystrophy. This provides important insights regarding the benefits of GLP-1R agonists for treating lipodystrophy, informing more widespread use to improve the health of individuals with this condition.

## Graphical Abstract

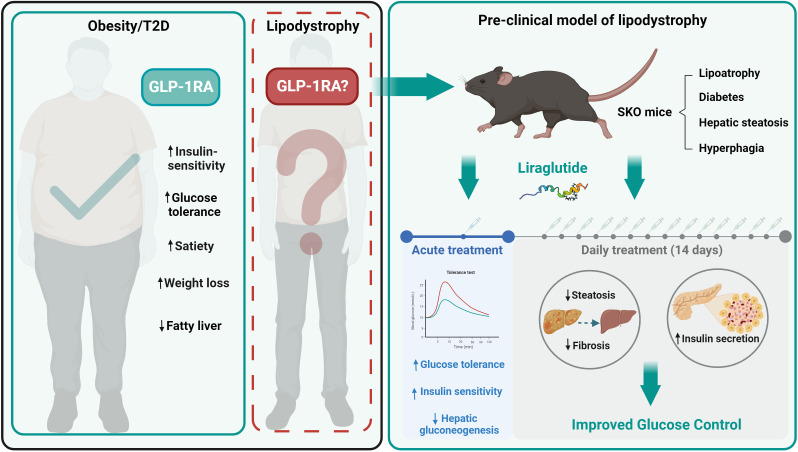

## 1 Introduction

Syndromes of lipodystrophy are rare disorders where reduced adipose mass or adipose tissue dysfunction leads to metabolic disease. Affected individuals are typically characterised by lipoatrophic diabetes, insulin resistance and hepatic steatosis (1–5). This is accompanied by a complex array of additional features depending on the nature of the lipodystrophy, making this a multisystem disease (2, 4, 6). Although rare, lipodystrophies are likely to be underdiagnosed, and recent estimates suggest a clinical prevalence of 1 in 20,000 (7). The most severe inherited forms of lipodystrophy are the congenital generalised lipodystrophies (CGL) in which adipose tissue may be almost entirely absent. Several more frequently occurring forms of familial partial lipodystrophy (FPLD) exist in which affected individuals may have apparently modest changes in absolute adipose mass but redistribution or dysfunction of adipose tissues (1, 2). Treatments for lipodystrophies are currently limited, and more effective therapies urgently needed (1). Metreleptin, which replaces the adipocyte hormone leptin, is the only specific treatment to alleviate the metabolic complications of lipodystrophy and can also offer significant improvements in quality of life (8–10). Metreleptin is highly effective in CGL but only effective in a subset of individuals with FPLD, typically those with low circulating leptin levels (9–11). In addition, metreleptin requires daily injection, which is painful in lipodystrophic individuals due to the paucity of subcutaneous adipose tissue, is costly and not available to patients in all countries. Hence, there is a clear need for new treatments especially lower cost, more accessible and orally available options.

Agonists of the glucagon-like peptide-1 receptor (GLP-1R) deliver significant beneficial effects both in treating type 2 diabetes and obesity (12, 13). Key effects of GLP-1R agonists are to produce improvements in insulin sensitivity, reduce food intake, reduce adiposity, and delay gastric emptying (12, 13). Type 2 diabetes and insulin resistance is most commonly associated with excess adiposity in overweight or obesity individuals (14, 15). However, the lipoatrophic diabetes observed in patients with lipodystrophies differs significantly from common type 2 diabetes in several regards, typically with significantly reduced adipose mass, dysregulation of adipose derived hormones including leptin and adiponectin, dyslipidaemia, more severe insulin resistance and significant hyperinsulinemia (2, 4, 6).

Reports of the effects of GLP-1R agonists in individuals with inherited lipodystrophies are currently limited to only 3 case studies, and these examine FPLD but not CGL patients (16–18). Moreover, with many CGL patients taking metreleptin currently, strong evidence of a therapeutic benefit would be needed before moving them to alternative medications. Hence, it is critical to gain insights specifically into the mechanisms underpinning any effects of GLP-1R agonists in preclinical models of lipodystrophy to better understand their use for treating patients with these conditions. For example, reducing adiposity further would not be a desired outcome for individuals with lipodystrophy, as the limited adipose tissue remaining is essential for health. As such, examining the effect of GLP-1R agonists in a model of lipodystrophy is required before widespread adoption of its use in patients.

Here we used a well validated *Bscl2*-null (seipin knockout) mouse model of CGL type 2 (CGL2) to examine the effectiveness of a GLP-1R agonist, in treating lipoatrophic diabetes, hepatic steatosis and hyperphagia in CGL to inform the adoption of this class of drugs for individuals with lipodystrophy.

## 2 Materials and methods

### 2.1 Animal studies

Seipin knockout (SKO) mice with germline disruption of the *Bscl2* gene were generated as previously described on a C57BL/6J background (19). Unless otherwise stated, mice had *ad libitum* access to water and food consisting of a standard rodent chow diet (CRM (P) 801722, Special Diets Services). Mice were group-housed by gender at 20-24°C, 45-65% humidity and exposed to a 12-hour light/12-hour dark period. Fed blood glucose levels were determined at 10:00 AM from tail punctures using a glucometer (AlphaTrak^®^ II, Zoetisus). Body composition (fat mass and lean mass) was measured using a EchoMRI-500 analyser (Zinsser Analytic). Tissues were rapidly dissected post-mortem and either snap-frozen in liquid nitrogen and stored at -70°C or fixed in 10% neutral buffered formalin for molecular analysis and histology, respectively. All procedures were carried out in accordance with the U.K. Animals (Scientific Procedures) Act 1986 and following local ethical approval at University of Aberdeen.

### 2.2 Treatment and tolerance tests

Male and female mice (22- to 25-week-old) were placed in clean cages and food withheld for five hours. Mice were randomly assigned to treatment and control groups. 0.2 mg/kg liraglutide (Tocris) was intraperitoneally injected to the treatment group 45 minutes before the tolerance tests (-45 min). Phosphate-buffered saline (PBS) was used as a vehicle and injected in control mice at the same time. Basal glucose levels (0 min) were determined by glucometer readings (AlphaTrak^®^ II, Zoetisus) from tail punctures. Mice then received an intraperitoneal bolus of 2 mg/g D-Glucose (Sigma-Aldrich) for glucose tolerance tests (GTT), 0.75 IU/kg of human insulin (Actrapid 100 IU/mL, Novo Nordisk) for insulin tolerance tests (ITT) or 2 g/kg pyruvate (Sigma-Aldrich) for pyruvate tolerance tests (PTT) as in (20). Blood glucose levels were then monitored at 15, 30, 60, 90 and 120 minutes. Mice had *ad libitum* access to water throughout the experiment. For the sub-chronic treatment, mice received a daily intraperitoneal injection of either 0.2 mg/kg liraglutide (Tocris) or vehicle (PBS) 30 minutes before lights out for 14 days.

### 2.3 Metabolic assessment

Mice were singly housed in cages automatically collecting food and water intake data (PhenoMaster, TSE Systems). Mice acclimatized to the cages for 3 days prior to measurements. Food intake and water consumption were measured over 8 consecutive days as in (20). Body weights were recorded daily.

### 2.4 Serum analysis

Blood was collected by cardiac puncture in SST™ Amber tubes (BD Microtainer^®^) from five-hour fasted mice. After 30 minutes at room temperature, samples were centrifuged at 12,000 x g for 10 minutes at 4°C and serum collected. Serum triglyceride contents were determined using the Triglyceride Liquid Assay (Sentinel Diagnostics) following manufacturer’s instructions. Serum insulin levels were measured using a mouse insulin ELISA kit (Mercodia).

### 2.5 Liver triglyceride assay

Frozen liver tissue samples were weighed (~100 mg) and homogenised by rapid agitation in 1 mL of phosphate-buffered saline with 1 mm diameter zirconia beads (BioSpec products, #11079110zx) using Precellys-Bertin homogeniser (Bertin Technologies). Liver lysates were then centrifuged at 12,000 x g for 10 minutes at 4°C. Triglycerides levels were determined from the supernatants using the Triglyceride Liquid Assay (Sentinel Diagnostics) following manufacturer’s instructions as in (21).

### 2.6 Gene expression

RNA was extracted from frozen liver tissues using the RNeasy mini kit (Qiagen), treated with DNase I (Sigma-Aldrich), then reverse-transcribed with M-MLV reverse transcriptase (Promega). Real-time quantitative PCR was carried out on the CFX384 Touch Real-Time PCR Detection System (BIO-RAD). Gene expression was normalised to the geometric mean of three stable reference genes (*Nono*, *Ywhaz* and *Hprt*). Sequences and details of primers and assay probes are provided in the **Supplementary Table S2**.

### 2.7 Histology, IHC and β-cell mass measurement

Liver tissue and pancreata were fixed in 10% neutral buffered formalin solution for 24 hours then subsequently embedded in paraffin. 5 µm-thick sections were stained with hematoxylin and eosin (H&E) or picrosirius red solution (Abcam #ab150681) following manufacturer’s protocol as previously described (22). For insulin staining, pancreatic sections were incubated overnight at 4°C with anti-insulin antibody (1:500, Clone L6B10, Cell Signalling). Chromogenic detection was performed with an HRP-conjugated mouse secondary antibody using 3,3’-diaminobenzidine (DAB, Vector Laboratories, #SK-4100) substrate. For β-cell area quantification, between 6 and 10 pancreata per gender and genotype and for each treatment were analysed. For each pancreas, 3 sections 150 μm apart from each other were stained for insulin. The total area occupied by insulin-positive cells was measured using QuPath software (23) and expressed as a percentage of the total pancreatic area. Researchers were blind to the group allocation and outcome assessment.

### 2.8 Statistical analyses

All the data are presented as mean ± SEM and analysed by unpaired two-tailed Student’s t-test or two-way ANOVA with Bonferroni or Tukey’s post-hoc test as appropriate using GraphPad Prism. P < 0.05 was considered as statistically significant.

## 3 Results

### 3.1 Liraglutide improves insulin and glucose tolerance in lipodystrophic mice

**Figure 1:**
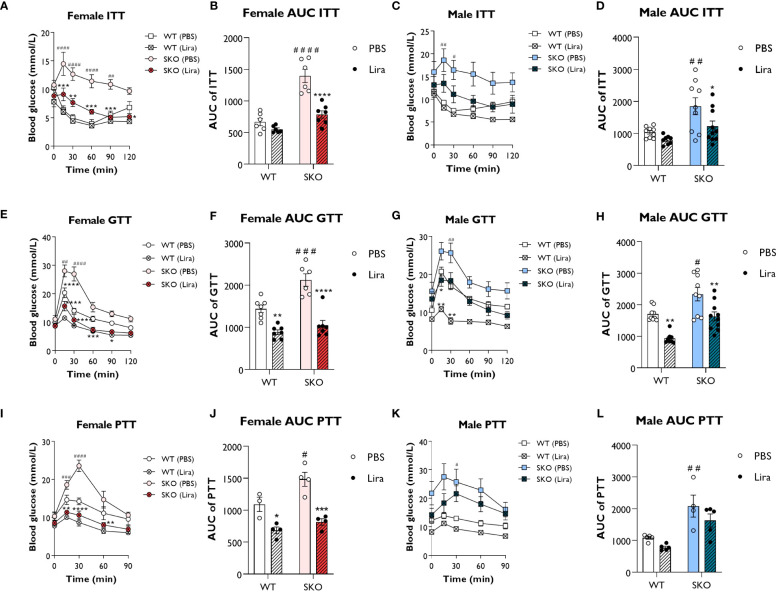
Liraglutide improves glucose tolerance and insulin sensitivity in mice with lipodystrophy. Male or female 22-week-old wild-type (WT) and seipin knockout (SKO) mice were intraperitoneally injected with phosphate-buffered saline (PBS) or 0.2 mg/kg liraglutide (Lira) prior to insulin tolerance tests (ITT) **(A–D)**, glucose tolerance tests (GTT) **(E–H)** and pyruvate tolerance tests (PTT) **(I–L)**. Areas under the curve (AUC) are shown for ITTs **(B, D)**, GTTs **(F, H)** and PTTs **(J, L)** in female and male mice as indicated (female n= 3-7, male n= 4-10). Data are presented as mean ± SEM; *p < 0.05, **p < 0.01, ***p < 0.001, ****p < 0.0001 (PBS versus liraglutide within same genotype); ^#^p < 0.05, ^##^p < 0.01, ^###^p < 0.001, ^####^p < 0.0001 [WT (PBS) versus SKO (PBS)].

We first investigated the acute effects of GLP-1 agonism in male and female seipin knockout (SKO) mice. Liraglutide (0.2 mg/kg) was intraperitoneally injected 45 min before performing insulin, glucose, and pyruvate tolerance tests (ITT, GTT and PTT respectively). As expected, SKO mice failed to respond to insulin during an ITT, consistent with severe insulin resistance (**Figures 1A–D**). Liraglutide treatment significantly improved insulin sensitivity in SKO mice compared to PBS-treated SKO controls. Area under the curve (AUC) analysis showed significant decrease in SKO-treated mice, returning to values similar to those observed in wild-type (WT) PBS-treated mice (**Figures 1B, D**). During a GTT, SKO mice showed impaired glucose clearance compared to WT littermate controls (**Figures 1E–H**), indicating glucose intolerance. Glucose clearance rates were significantly improved in liraglutide-treated female SKO mice and were similar to those observed in liraglutide-treated WT mice (**Figures 1E, F**). Glucose clearance in liraglutide-treated male SKO returned to levels observed in WT PBS-treated controls (**Figures 1G, H**). As reported previously, liraglutide significantly improved glucose tolerance in WT mice (24–26). A PTT is used as an indirect measure of gluconeogenesis. In response to pyruvate injection, significantly greater increases in blood glucose levels were observed in female SKO mice injected with PBS compared with similarly treated WT controls. This was completely abolished by liraglutide treatment, leading to blood glucose levels lower than those observed in WT PBS-treated mice (**Figures 1I, J**). Liraglutide treatment also partially prevented this glucose excursion in male SKO mice, but this failed to reach statistical significance (**Figures 1K, L**).

Collectively, these data show that acute liraglutide treatment significantly improves glucose tolerance, insulin sensitivity and impaired hepatic gluconeogenesis in SKO mice, with the greatest effects being observed in female SKO mice.

### 3.2 Effect of liraglutide on food and water intake, body composition and glycaemia

**Figure 2:**
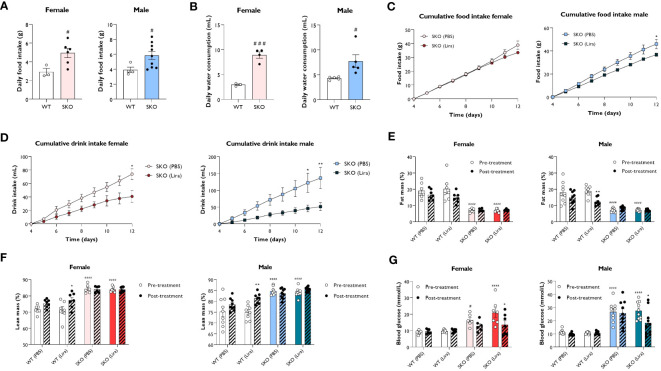
Effect of liraglutide on food and water intake, body composition and glycaemia. Average daily food intake **(A)** and water consumption **(B)** for 22-week-old singly-housed wild-type (WT) and seipin knockout (SKO) mice measured over three to seven days. Female and male WT and SKO mice were intraperitoneally injected once daily with phosphate-buffered saline (PBS) or 0.2 mg/kg liraglutide (Lira) for 14 days as indicated and food intake **(C)**, water consumption **(D)**, fat mass **(E)**, lean mass **(F)** and non-fasted blood glucose levels **(G)** were determined (female n= 3-7, male n= 4-10). Data are presented as mean ± SEM; *p < 0.05, **p < 0.01 (PBS versus liraglutide within same genotype); ^#^p < 0.05, ^###^p < 0.001, ^####^p < 0.0001 [WT (PBS) versus SKO (PBS)].

In addition to its insulinotropic action, GLP-1 has also been shown to reduce food intake and slow gastric emptying (27). To establish metabolic changes induced by liraglutide in mice with CGL, WT and SKO mice were given daily intraperitoneal injections of PBS (vehicle) or 0.2 mg/kg liraglutide for 14 days. We examined the effect of liraglutide administration on food intake, water consumption, body composition and non-fasted blood glucose levels, assessed over 8 consecutive days. In accordance with clinical features observed in CGL2 patients, SKO mice were hyperphagic (**Figure 2A**), attributable to the low circulating levels of leptin (28–32). Water intake was also significantly elevated in SKO mice when compared to WT mice (**Figure 2B**), which is consistent with polydipsia, a recognised feature of diabetes (33). The 2-week liraglutide administration did not significantly alter food intake in female SKO mice although there was a slight decrease around day 12 of treatment (**Figure 2C**). Liraglutide had a more pronounced effect in male SKO mice where it significantly decreased food intake by day 12 when compared to PBS-injected SKO mice (**Figure 2C**). SKO mice injected with liraglutide also showed a progressive normalisation of water consumption with a 45% and 62% reduction by day 12 in female and male SKO mice, respectively (**Figure 2D**). This likely reflects an anti-diabetic treatment effect of liraglutide on SKO mice. As shown previously (19, 28, 29, 34), lipodystrophic SKO mice display significantly reduced fat mass (**Figures 2E**; **Supplementary Figure S1A**) and increased lean mass (**Figure 2F**; **Supplementary Figure S1B**) relative to WT mice. When comparing metabolic parameters pre and post the 2-week treatment, we observed that liraglutide significantly lowered fat mass in male WT mice, however similar changes failed to reach statistical significance in female WT mice (**Figure 2E**). This was associated with a significant increase in lean mass in both male and female mice WT mice (**Figure 2F**). In contrast, body composition of SKO mice was not affected by liraglutide or vehicle treatment (**Figures 2E, F**; **Supplementary Figures S1A, B**). We also observed increased tissue weights for heart and kidneys in female but not male SKO mice, consistent with previously reported organomegaly in this model (35). This was not altered by treatment with liraglutide for 14 days (**Supplementary Figures S1C, D**) Finally, blood glucose levels were examined in the non-fasted state before and after 10 days of treatment. Liraglutide did not alter blood glucose levels in WT mice, which remained around 10 mmol/L. Prior to the study, SKO mice were hyperglycaemic with blood glucose levels of approximately 16.4-21.4 mmol/L and 26.7-27.5 mmol/L for female and male SKO mice, respectively (**Figure 2G**). PBS-treated SKO mice had comparable blood glucose levels pre- and post-treatment. However, SKO hyperglycaemia was significantly lowered by liraglutide treatment with both male and female SKO mice displaying a ~35% reduction in blood glucose concentrations (**Figure 2G**).

These results demonstrate that liraglutide efficiently reduced polydipsia and hyperglycaemia without altering adiposity in mice with generalised lipodystrophy.

### 3.3 Liraglutide improves hepatic steatosis and fibrosis development in lipodystrophic mice

**Figure 3:**
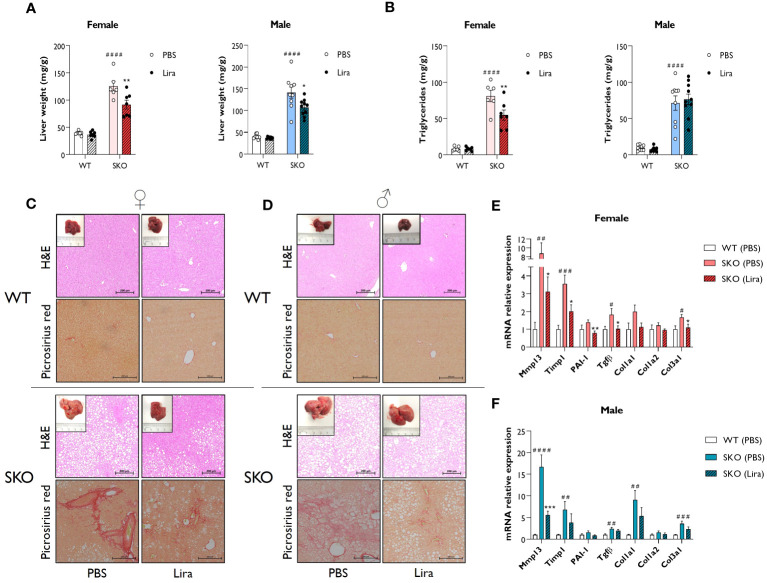
Liraglutide improves hepatic steatosis and fibrosis in mice with lipodystrophy. Male and female wild-type (WT) and seipin knockout (SKO) mice were intraperitoneally injected once daily with phosphate-buffered saline (PBS) or 0.2 mg/kg liraglutide (Lira) for 14 days. **(A)** Liver weight normalised to mice body weight. **(B)** Levels of hepatic triglycerides. **(C, D)** Representative images of hematoxylin and eosin (H&E) and picrosirius red-stained paraffin-embedded liver sections along with representative photographs of macroscopic liver appearance in female **(C)** and male **(D)** mice. Bars denote 200 µm. **(E, F)** Expression levels of a panel of fibrosis markers genes in WT mice and SKO mice treated with PBS or liraglutide. Gene expression was normalised to three reference genes (*Hprt*, *Nono* and *Ywhaz*) (female n= 6-7, male n= 8-10). Data are presented as mean ± SEM; *p < 0.05, **p < 0.01, ***p < 0.001 (PBS versus liraglutide within same genotype); ^#^p < 0.05, ^##^p < 0.01, ^###^p < 0.001, ^####^p < 0.0001 [WT (PBS) versus SKO (PBS)].

Due to an inability to appropriately store lipids, individuals with lipodystrophies typically develop metabolic dysfunction-associated steatotic liver disease (MASLD) which often progresses to hepatic steatosis and fibrosis. Recapitulating the human condition, SKO mice displayed significantly enlarged livers when compared to WT controls (**Figure 3A**) (30, 31, 36). This was associated with the substantial accumulation of lipids, principally triglycerides, within this tissue (**Figure 3B**). Daily liraglutide treatment for 14 days significantly improved liver health in SKO mice. Specifically, liver weights were decreased by 24% in male and 27% in female SKO mice (**Figure 3A**). Hepatic triglyceride content was significantly reduced by 32% in female SKO mice (**Figure 3B**) but was not different between PBS and liraglutide-treated male SKO mice (**Figure 3B**). Consistent with these findings, H&E staining of liver sections revealed significant lipid accumulation in SKO mice that was considerably reduced by liraglutide treatment in female SKO mice (**Figure 3C**) but this was not evident in liraglutide-treated male SKO mice (**Figure 3D**). Picrosirius red staining showed substantial fibrosis in both male and female SKO mice when compared to WT mice (**Figures 3C, D**). Administration of liraglutide qualitatively reduced the presence of collagen fibres in livers of SKO mice (**Figures 3C, D**). To quantitatively examine hepatic fibrosis in SKO mice, the expression of pro-fibrogenic markers were analysed. In agreement with histological staining, mRNA levels of several markers, including *Mmp13*, *Timp1*, *Tgfβ* and *Col3a1* were significantly upregulated in both male and female PBS-treated SKO mice when compared to the PBS-treated WT group (**Figures 3E, F**). The levels of *Mmp13*, *Timp1*, *PAI-1*, *Tgfβ* and *Col3a1*, were significantly reduced by liraglutide treatment in female SKO mice, consistent with the histological observations (**Figure 3E**). *Mmp13* expression was also significantly lower in liraglutide-treated male SKO mice (**Figure 3F**). Moreover, there was a trend toward a liraglutide-induced decrease in the expression of other markers of fibrosis examined in both male and female SKO mice, although these failed to reach statistical significance (**Figures 3E, F**). Liraglutide treatment did not alter expression of these genes in WT mice (data not shown).

Taken together, these data indicate that liraglutide treatment significantly improves hepatic steatosis and liver fibrosis in female SKO mice. These effects were less pronounced in male SKO mice, although beneficial effects were still observed on hepatomegaly and fibrosis.

### 3.4 Liraglutide promotes insulin secretion in lipodystrophic mice

**Figure 4:**
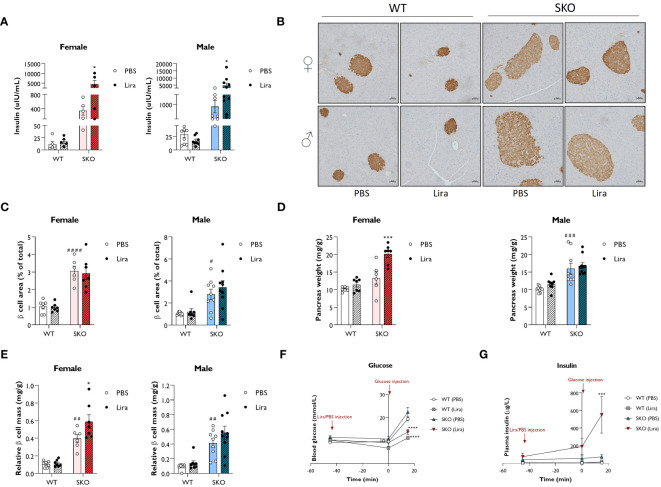
Liraglutide promotes insulin secretion in mice with lipodystrophy. Wild-type (WT) and seipin knockout (SKO) female and male mice were treated daily with 0.2 mg/kg liraglutide (Lira) for 14 days. Phosphate-buffered saline (PBS) was used as a vehicle control. **(A)** Effects of daily injections of liraglutide on serum insulin levels in female and male WT and SKO mice. **(B)** Representative images of insulin staining in pancreatic sections. **(C)** Quantification of insulin-stained pancreatic sections. Results are expressed as a percentage of the total pancreatic area occupied by β cells. **(D)** Pancreas weight normalised to body weight. **(E)** Relative β cell mass to pancreas weight normalised to body weight (female n= 5-7, male n= 8-10). **(F, G)** Acute effects of a single injection of 0.2 mg/kg liraglutide on both blood glucose **(F)** and plasma insulin **(G)** in male and female WT and SKO mice (n= 3-8). Data are presented as mean ± SEM; *p < 0.05, ***p < 0.001, ****p < 0.0001 (PBS versus liraglutide within same genotype); ^#^p < 0.05, ^##^p < 0.01, ^###^p < 0.001, ^####^p < 0.0001 [WT (PBS) versus SKO (PBS)].

Liraglutide, and GLP-1 agonists more broadly, have been shown to improve β cell function by promoting insulin secretion, β cell survival and β cell proliferation (12, 13, 37–39). Therefore, we examined whether these mechanisms contribute to the beneficial effects of liraglutide on glucose tolerance and hepatic steatosis observed in SKO mice. Fasting circulating insulin levels were ~30-fold higher in male and female SKO mice when compared to WT control mice. Two weeks of daily injections with 0.2 mg/kg liraglutide further increased insulin levels in both male and female SKO mice (**Figure 4A**). To evaluate whether this increase in insulin secretion could be attributed to an increase in β cell mass and/or an enhanced secretory capacity of the islets, immunohistochemical analysis of the pancreas and glucose-stimulated insulin secretion (GSIS) assay were performed. Insulin staining of pancreatic sections were consistent with changes in insulin secretion observed in **Figure 4A** with an increase in β cell mass in SKO mice relative to WT mice (**Figure 4B**). As observed in other insulin-resistant mouse models, this increase in β cell mass is likely to act as a compensatory mechanism to counteract the insulin resistance in SKO mice. Detailed morphometric analysis of the pancreas revealed a 3-fold increase in pancreatic β cell area in male and female SKO mice when compared to WT control mice (**Figure 4C**). β cell area was defined as the percentage of the total pancreatic area that was occupied by insulin-positive cells. While liraglutide- and PBS-treated SKO mice displayed comparable density of β cell area as a proportion of pancreatic area (**Figure 4C**), liraglutide treatment significantly increased pancreas weight in female SKO mice leading to an increase in total β cell mass per animal when normalised to body weight (**Figures 4D, E**). Male SKO mice displayed significantly enlarged pancreases when compared to WT mice, but this was not altered by liraglutide treatment (**Figure 4D**). As a result, total β cell mass was not significantly changed by liraglutide treatment male SKO mice, although there was a similar trend to that observed in female mice (**Figures 4D, E**). To interrogate whether liraglutide improved β cell function, we assessed GSIS *in vivo* (**Figures 4F, G**; **Supplementary Table S1**). Mice were fasted for five hours, and blood samples collected before and after administration of liraglutide and D-glucose to assess circulating blood glucose and insulin levels. SKO mice exhibited fasting blood glucose comparable to their WT counterparts (**Figure 4F**; **Supplementary Table S1**). Fasting insulin levels were elevated in SKO mice when compared to WT controls but to a similar extent between vehicle- and liraglutide-treated groups (**Figure 4G**; **Supplementary Table S1**). Administration of liraglutide, in the absence of glucose challenge, modestly decreased glucose levels in WT mice but had no effect on SKO mice glucose levels. Plasma insulin concentrations increased during euglycemia in liraglutide-treated SKO mice, independently of any glucose stimulus. In response to a glucose bolus, a 2-fold increase in blood glucose levels was rapidly observed in WT and SKO mice treated with vehicle, with minimal fluctuation in plasma insulin levels (**Figures 4F, G**; **Supplementary Table S1**). Liraglutide markedly reduced these glycaemic excursions in both WT and SKO mice, and this occurred concomitantly with an increase in insulin secretion (**Figure 4G**; **Supplementary Table S1**). Although variable between mice, SKO mice administered with liraglutide released very high levels of insulin in response to glucose, 8 times higher than those of PBS-treated SKO mice and 40 times higher than those of liraglutide-treated WT mice.

Together, these data illustrate that daily injections of liraglutide strongly stimulate insulin secretion in lipodystrophic mice. Not only does liraglutide appear to modulate β cell mass, but it also enhances the capacity of islets of SKO mice to secrete insulin.

## 4 Discussion

The severe insulin resistance, hepatic steatosis, dyslipidaemia and hyperphagia that characterises lipodystrophy makes effective treatment challenging and limits the effectiveness of commonly used diabetes therapies (1–3, 40, 41). Recombinant leptin therapy is currently the only dedicated therapy for generalised lipodystrophy but is effective only in some cases of partial lipodystrophy. New effective therapies for lipodystrophy patients are therefore a clinical imperative (1, 3, 5, 41–43). Although GLP-1R agonists are widely used for common forms of type 2 diabetes, there are only three case study reports of their use in patients with lipodystrophy and few insights regarding their effects (16–18). Here we demonstrate that the GLP-1R agonist liraglutide is highly effective at improving multiple aspects of metabolic disease in a mouse model of generalised lipodystrophy. SKO mice recapitulate key features of generalised lipodystrophy seen in individuals with disruption of the corresponding gene, *BSCL2* (19, 28–30, 34). This includes insulin resistance, glucose intolerance, hepatic steatosis, very low adiponectin and leptin levels and hyperphagia, although not the hypertriglyceridemia observed in CGL2 patients. As such, SKO mice provide a valuable model to examine potential therapies for lipodystrophies. This is particularly the case given the lack of murine models accurately mimicking more frequently occurring partial lipodystrophies (2, 4). As noted above, metreleptin is ineffective in many patients with partial lipodystrophy and so this patient group is particularly in need of new treatment options (1, 5, 41, 42). One should be cautious extrapolating from our mouse SKO model of CGL2 to partial lipodystrophy. However, as liraglutide was effective without functional adipose tissue present in our SKO mice, this implies it may be effective in partial lipodystrophy where adipose tissue is dysfunctional or reduced. Indeed, the case reports that exist do suggest that GLP-1R agonists can be beneficial in individuals with partial lipodystrophy (16–18). However, more comprehensive clinical trials will be needed to confirm their effectiveness and if different subtypes of partial lipodystrophy respond differently to this class of drugs.

The data presented indicate that liraglutide improves lipoatrophic diabetes in SKO mice, with both improvements to insulin sensitivity and enhanced glucose regulated insulin release. Liraglutide treatment also improved fatty liver in SKO mice, with reduced hepatomegaly, steatosis, markers of fibrosis and evidence of acute improvements of gluconeogenesis regulation in the PTT assays. It has been noted that there is a need to better stratify patients with fatty liver disease to more accurately target appropriate treatments and that lipodystrophy associated fatty-liver disease should be considered distinct from other forms (44). As with other aspects of this study, we believe the work presented provides important new insights regarding exactly how fatty liver disease in lipodystrophy may respond to GLP-1R agonist treatment. Given the severe lipodystrophy in our mouse model, our data also reveal that GLP-1 receptor activation can improve insulin sensitivity and metabolic health independent of adipose tissue, providing additional novel mechanistic insight.

Modest effects of liraglutide treatment were observed on food intake in SKO mice, with significantly reduced food intake in male SKO mice but not in females. This could be related to very low leptin levels in this model of CGL. The GLP-1R agonist exenatide has been shown to have little effect on food intake in leptin deficient mice and leptin receptor null rats, whilst leptin can strongly enhance the anorectic effects of exenatide in the latter (45, 46). We did not examine whether liraglutide altered serum levels of leptin or adiponectin in our model. The levels of both are extremely low in our SKO mice, consistent with the fact that we are unable to identify and dissect any white adipose tissue (WAT) depots (22). For this reason, we ascribe the fat mass signal in our body composition data to ectopic lipid accumulation. We do observe a small residual brown adipose tissue (BAT) depot in our SKO mice (**Supplementary Figure S1E**). However, we see no alteration in this, nor rescue of any visible WAT depots, with liraglutide treatment. Therefore, we predict that adipokine levels are unlikely to be changed by liraglutide treatment, although this will be interesting to examine in future studies.

Related to this, it will be important to determine whether GLP-1R agonists could reduce appetite in individuals with CGL and FPL patients with low leptin. It may be that in these patients, leptin supplementation is required to fully normalise appetite control but that GLP-1R agonists would permit a much lower dose of leptin to achieve this. In addition, semaglutide has been shown to have greater appetite suppressive effects than liraglutide in overweight individuals and so may also be better at suppressing hyperphagia in lipodystrophy (12). In a similar manner, the increased insulin secretion in our liraglutide treated SKO mice indicate that GLP-1R agonists could drive greater endogenous insulin production in lipodystrophy patients and thereby reduce the requirements for high dose insulin injections that are often used by these individuals.

Another key potential advantage of GLP-1R agonists for lipodystrophy patients is the availability of both long acting and oral GLP-1R agonists (12, 13). A significant difficulty for patients is the need for injection of insulin or leptin and the associated pain due to the paucity of subcutaneous adipose tissue. Orally effective treatments for this condition would reduce pain and therefore improve quality of life for patients and is a considerable potential benefit of available oral GLP-1R agonists.

Despite clear advantages to GLP-1R agonist use in lipodystrophy presented here, some patients with lipodystrophy also develop pancreatitis. A recent systematic review of case reports of adverse events linked to GLP-1R agonists highlighted cases of pancreatitis associated with liraglutide and exenatide, although none with semaglutide (47). However, as this was a review of case reports it does not provide evidence of risk. Long term studies of liraglutide in rodents and primates have not revealed increased pancreatitis risk (48–50). Moreover, systematic reviews and meta-analyses of randomised controlled trials have not shown increased incidence of pancreatitis in patients with type 2 diabetes treated with liraglutide, exenatide or semaglutide (51–53). Appropriate monitoring will be necessary for GLP-1R agonists use in the treatment of patients with lipodystrophy.

Our study suggests that GLP-1R agonists improve insulin sensitivity, glycaemic control, steatosis, and pancreatic beta cell function in CGL and, likely, in lipodystrophies more broadly. These agents may prove effective alone or may permit significant reductions in the dosage requirement for existing treatments such as metreleptin and insulin. As the first study to examine in detail the effects of GLP-1R agonists in a pre-clinical model of lipodystrophy, we provide a valuable basis for future patient studies to ultimately relieve the suffering of these patients.

## Data availability statement

The original contributions presented in the study are publicly available. This data can be found here: The University of Aberdeen Research Portal, DOI: 10.20392/aef8fe2e-5180-4693-b187-750dfecf5e9e.

## Ethics statement

The animal study was approved by Animal procedures were approved by the University of Aberdeen Ethics Review Board. The study was conducted in accordance with the local legislation and institutional requirements.

## Author contributions

AR: Conceptualization, Writing – original draft, Formal analysis, Investigation, Methodology, Writing – review & editing. GM: Formal analysis, Investigation, Methodology, Writing – review & editing, Writing – original draft. NS: Formal analysis, Investigation, Writing – review & editing, Writing – original draft. WH: Formal analysis, Writing – review & editing, Resources, Writing – original draft. LH: Formal analysis, Resources, Writing – review & editing, Methodology, Project administration, Writing – original draft. JR: Project administration, Resources, Conceptualization, Funding acquisition, Supervision, Writing – original draft, Writing – review & editing.
